# Mechanisms Explaining Nursery Habitat Association: How Do Juvenile Snapper (*Chrysophrys auratus*) Benefit from Their Nursery Habitat?

**DOI:** 10.1371/journal.pone.0122137

**Published:** 2015-03-24

**Authors:** Darren M. Parsons, Crispin Middleton, Keren T. Spong, Graeme Mackay, Matt D. Smith, Dane Buckthought

**Affiliations:** 1 NIWA, Auckland, New Zealand; 2 NIWA, Ruakaka, New Zealand; University of Auckland, NEW ZEALAND

## Abstract

Nursery habitats provide elevated survival and growth to the organisms that associate with them, and as such are a crucial early life-stage component for many fishes and invertebrates. The exact mechanisms by which these benefits are afforded to associated organisms, however, are often unclear. Here we assessed potential explanations of the nursery function of structurally complex habitats for post-settlement snapper, *Chrysophrys auratus*, in New Zealand. Specifically, we deployed Artificial Seagrass Units (ASUs) and used a combination of video observation, netting and diet analysis of associated post-settlement snapper as well describing potential prey within the micro-habitats surrounding ASUs. We did not observe any predation attempts and few potential predators, suggesting that for snapper the nursery value of structurally complex habitats is not as a predation refuge. The diet of post-settlement snapper mostly consisted of calanoid and cyclopoid copepods, which were most commonly sampled from within the water column. Nearly all suspected feeding events were also observed within the water column. When considering the velocity of water flow at each ASU, plankton sampling revealed a greater availability of copepods with increasing current strength, while netting and video observation demonstrated that the abundance of snapper was highest at sites with intermediate water velocity. This study highlights that the interaction between water flow and food availability may represent an important trade-off between energy expenditure and food intake for post-settlement snapper. Structurally complex habitats may mediate this relationship, allowing snapper to access sites with higher food availability while reducing swimming costs. This mechanism may have broader relevance, potentially explaining the importance of estuarine nursery habitats for other species.

## Introduction

Many fish species occupy nursery habitats during critical early life phases, before moving to separate adult habitats [1–4]. These habitats are termed nurseries because they provide a disproportionately high supply of recruits to the adult population [1]. This definition implies that nursery utilising species are constrained to a set of habitats during the juvenile life phase. Such a situation may only come about if a strong benefit is afforded by that habitat. Although determining the mechanisms of benefit is difficult, it is generally believed to involve protection from predation and/or an increased provision of food [3], because nursery function is linked to increased survival and growth [1].

Snapper, *Chrysophrys auratus* (= *Pagrus auratus*) (F. Sparidae), are a recreationally and commercially important fish species that is abundant in northern New Zealand [5]. High abundances of post-settlement juvenile snapper (<60 mm Fork Length (FL)) have only been observed in association with structurally complex habitats within estuaries, such as seagrass [5]. An explanation for this tight association with shallow water structurally complex habitats, however, is not apparent. Direct and indirect observations of predation are rare and the importance of predation on recruits and juveniles is unknown [5]. Furthermore, when post-settlement snapper associated with seagrass beds are approached by predators, they generally swim away from the threat and often away from the seagrass as well (D. Parsons pers. obs.). This is not the expected response of fish that utilise structurally complex habitats as a refuge from predation. While larger snapper (1+ and greater) predominantly prey on benthic items [6], an ontogetic shift in diet could occur as pelagic crustaceans may form the majority of the diet of post-settlement snapper [7] (especially in estuaries with low turbidity [8]). If the majority of food is acquired planktivorously, then it is not immediately apparent how structurally complex habitats would serve to increase food intake.

A potential mechanism explaining the association between post-settlement snapper and structurally complex habitats may be connected to the tidal flows in estuaries where this life stage is abundant. Assuming that post-settlement snapper are predominantly planktivorous [7,8], the combination of structurally complex habitat and fast water flow may confer benefits through an increased flux of food and the provision of an energetic refuge from water currents. A similar mechanism has been established for salmonids inhabiting streams [9], whereby optimal stream positions are determined by low water velocity to minimise energy expended during swimming, but adjacent to high water velocity to maximise energy gain from invertebrate drift.

Here we conducted an investigation to test the above flow-refuge / food maximisation hypothesis. We utilised Artificial Seagrass Units (ASUs) to standardise the size, shoot density, height-above-bed, and positions of individual ‘seagrass’ patches within estuaries. We sampled sites covering a range of tidally driven water velocities, and collected observational rather than truly experimental data. Our goal was to compare and contrast the diet of post-settlement snapper with potential diet items (sampled from the sediment, amongst ASU blades and in the water column) and to compare the abundance and condition of snapper occupying ASUs at these sites. We also sought to make behavioural observations to determine how the ASU patches are utilised, where the feeding activities of post-settlement snapper were focused, and whether predation events or threats were important.

## Materials and Methods

**Fig 1:**
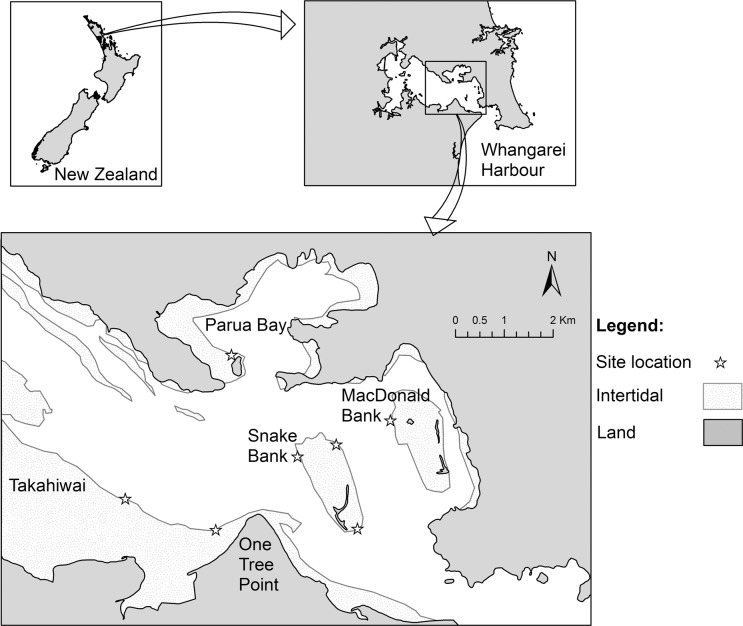
Map of Whangarei Harbour. Intertidal areas and the location of sampling sites indicated by light grey shading and starts respectively. New Zealand and the location of Whangarei Harbour within east-northland inset.

Experiments were conducted within Whangarei Harbour, northeastern New Zealand (Fig. 1). Whangarei Harbour is a drowned river valley of more than 100 km^2^, although only c. half of this area is flooded at low tide [10]. The harbour contains many banks and flats, which offer large areas of shallow subtidal seabed suitable for conducting experiments on juvenile fishes. While extensive seagrass beds were formerly present within the harbour, these largely disappeared during the last half of the 20^th^ century [11]. Recently, extensive seagrass recovery has taken place along the southern shoreline of the middle harbour (D. Parsons pers. obs.). We selected sites within Whangarei Harbour (Takahiwai: 174.424° E, 35.816° S; Parua Bay: 174.447° E, -35.789° S; Snake Bank West: 174.462° E, -35.808° S; Snake Bank East: 174.471° E, -35.805° S; Snake Bank South: 174.471° E, -35.805° S; MacDonald Bank: 174.483° E, -35.800° S; Fig. 1) using water velocity estimates provided from a previously developed three dimensional, flexible sized mesh, hydrodynamic and dispersion model [12]. This model incorporated extensive bathymetric data, and was verified with water level, salinity and current speed observational data. The six sites selected covered a wide range of tidal velocities, with mean values from 0.045 to 0.287 ms^-1^. All of these sites offered shallow, subtidal sediment flats that were devoid of natural structure, had similar levels of turbidity and were remote from access points that may elevate levels of human interference. In December 2012 (the time of peak snapper spawning [5]) we deployed four 1.8 m × 1.8 m ASUs at a water depth of 0.4 m at low tide at each site. ASUs were separated by 20 m. Previous investigations demonstrate that post-settlement snapper are resident to patches separated by c. 10 m [13], so we treated the four patches per site as independent replicates. Each ASU was constructed on a metal grid, with individual plastic seagrass plants (consisting of a rigid wire stem with multiple blades ~30 cm length) attached to the grid with cable ties. All ASUs had the same shoot density (216 plants; equivalent to 1820 blades m^−2^). This density was the same as the ‘high’ density treatment used by Parsons et al. [13], and corresponds to the upper range of subtidal seagrass densities in northeastern New Zealand [14]. A variety of different sample types were collected from the six sites described above, from mid-February to mid-March 2013. This is the time of year when post-settlement snapper are most abundant within estuarine nursery habitats [13]. All collections were conducted under NIWAs special permit (542) from the Ministry for Primary Industries and no protected species were sampled. In accordance with New Zealand’s Animal Welfare Act 1999 our collections did not require approval from the NIWA Animal Ethics Committee as all animals collected were killed (placed in containers with preservative or put on ice) without any manipulation.

### Sample collection and processing

#### Video footage

To gain insight into how post-settlement snapper utilise structurally complex habitats, we deployed cameras at each site between the 19^th^ of February and 19^th^ of March 2013. We used GoPro Hero 3 cameras, held in place with 15 cm high steel frames. Cameras were placed close enough to each ASU so that individual snapper (c. 60 mm FL) could be observed and so that roughly half the field of view encompassed bare sediment in front of the ASU and the other half encompassed the ASU itself. Cameras were placed on the up current edge of ASUs, although this sometimes changed if the deployment period included a high or low tide and subsequent switch in tidal direction. Cameras were deployed for about three hours (between 0700 and 1900 hrs), with some variability due to battery life variation. Replication at each site was limited by the four ASUs present. A total of twelve separate camera deployments, however, were conducted across the four ASUs at each site over eleven days to sample a greater range of time periods and tidal states. A total of 90 hours of video footage was collected across all ASUs.

Video footage was analysed using a sub-sampling procedure. This provided discrete time periods within which response variables could be categorised, reduced the total amount of video footage that needed to be analysed, and allowed us to combine data from multiple camera deployments into individual replicate means. After preliminary viewing of a series of videos we decided that a two minute time period provided a balance between being too short and potentially missing the presence of fish, and being too long and incorporating a high level of variation in abundance and behaviours. We followed a procedure of viewing two minutes and then skipping four.

While viewing footage we recorded four types of responses: (1) Presence/absence of post-settlement snapper. While we were not able to determine the exact length of snapper present in the video footage, the seagrass blades could be used for scale. During February/March there should be a c. 6 cm or c. 100% difference in the length of post-settlement and 1+ snapper [15], providing adequate resolution to categorise snapper into age classes. While snapper larger than the post-settlement stage (i.e. 1+ or greater) were observed around the ASUs on the video footage, they were not the focus of this investigation as they are not dependent on these nursery habitats. (2) Physical location of post-settlement snapper relative to the seagrass edge. If post-settlement snapper were present we also categorised their physical location relative to the ASU. The categories that we used were “within seagrass” if snapper could be seen amongst or moving into or out of the seagrass blades, “near seagrass” if snapper were within 30 cm of the seagrass, either above or next to it, “near seabed” if snapper were more than 30 cm away from the seagrass but within 10 cm of the seabed, and “away from seagrass or seabed” if snapper were not within 30 cm of the seagrass or 10 cm of the seabed. While some variability may occur in the location of snapper during a two minute period, we categorised this response variable to best match the location where the majority of the post-settlement snapper were observed throughout the time period. (3) Presence/Absence and location of feeding events. If post-settlement snapper were present we recorded whether feeding could be observed and if so, the location of feeding behaviour. While we could not confirm that feeding was actually occurring, we characterised suspected feeding events as post-settlement snapper making sudden short distance (a few cm) movements often associated with a mouth opening and/or a flexed operculum. These behaviours are consistent with the aspects of feeding behaviour described for other fishes consuming evasive prey [16–18]. The location of these feeding events was categorised as being either “amongst seagrass blades”, on the “sediment”, or in the “water column”. (4) Presence/Absence of predators and predation event occurrence/absence. Potential predators of post-settlement snapper were considered as medium to large sized (c. 25 cm FL or greater) carnivores that have a piscivorous component to their diet.

In addition to recording the above response variables we also recorded water visibility and camera obstructions. These were used to filter the data set. For example, if water visibility dropped below 1 m or if an object (floating debris) obstructed the camera lens, the footage was excluded from the analysis.

#### Zooplankton

Zooplankton were sampled to describe the potential diet items of post-settlement snapper that may occur within the water column. Sampling was undertaken during the day on six different days, at the six sites where ASUs were deployed and across the time period when post-settlement snapper are most abundant in this habitat type (mid—February to mid—March). Water samples were collected with a plankton pump that consisted of a petrol powered impellor pump connected to a 6.5 cm diameter hose that was long enough to reach the seabed (2–4 m depth). The hose was lowered to the seabed and held in place with an attached metal pole. A 50 cm long metal arm protruded below the end of the hose. This ensured that the water sample was always taken at a consistent elevation above the seabed. We chose an elevation of 50 cm as we deemed this would allow us to obtain near seabed water samples, which is where within water column feeding of post-settlement snapper would be likely to occur. The pump was then run until a 1200 l water sample had passed through a 250μm sieve (measured by repetitively filling a 20 l bucket). Samples were washed off the sieve and fixed in a 10% formalin solution before being preserved in 70% ethanol. A total of six zooplankton samples were collected from haphazardly selected locations immediately adjacent to the ASUs at each site. All invertebrates were then sorted, counted and identified down to the lowest practical taxonomic level.

#### Juvenile fish, gut contents, sediment and seagrass

At the beginning of April, before post-settlement snapper leave estuarine nursery habitats, a final collection of samples was undertaken. This consisted of sampling the sediment and artificial seagrass blades for potential diet items of post-settlement snapper, as well as sampling the fish communities associated with the ASUs themselves. To address the possibility that different invertebrate taxa might be available to juvenile snapper amongst natural seagrass we also took samples of potential diet items from patches of natural subtidal seagrass. Fish communities were sampled using the netting method described in detail by Parsons et al. [13]. Briefly, this sampling method is conceptually similar to a ‘brush and dust pan’, in that juvenile fish are ‘swept’ off the ASU with the brush (a fine meshed net weighted at the bottom with chain) and herded into the dustpan (a triangular fine meshed net with a floor). Sampling was conducted in wading depth water around low tide and performed twice on each ASU before we haphazardly selected another ASU at a site to be sampled. Fish captured during this process were immediately bagged and labelled according to the particular site and ASU and frozen. These fish were later identified to the species level, counted and the total length or FL measured to the nearest mm. For juvenile snapper, the focus of this investigation, each individual was also weighed (± 0.01 g). While the efficiency of this sampling method has not been established, in combination with the video observations described above this should provide a robust means to interpret snapper abundance.

A sub-sample of the juvenile snapper captured at each site (n = 60) also underwent additional processing so that they could be used to quantify diet. These fish received an injection of a 10% formalin solution (salt water buffered) into the gut cavity upon capture and were then immersed into the same solution. Gut contents were later obtained by making a ventral incision, removing and opening the fore and hindgut and washing the sample into a vial using a 70% ethanol mixture.

The sediment immediately adjacent to the ASUs at each site was sampled by taking a scrape of the upper c. 1 cm of sediment using a 100 ml sample jar. A total of five sediment scrapes were taken from each site and fixed in a 10% formalin solution. The volume of each sample was then measured before the sample was sieved on 250 μm mesh and preserved in 70% ethanol and 0.2% Rose Bengal stain. Invertebrates were then manually removed from the remaining sample and identified to the lowest taxonomic level practicable.

Artificial and natural seagrass blades were sampled by placing a 1 l jar over an individual plant and then detaching the plant from the ASU or seabed while it remained inside the jar. Three seagrass blade samples were taken from the ASUs at each site. Natural subtidal seagrass samples were taken from One Tree Point (174.444° E, 35.821° S; Fig. 1), where we haphazardly selected three patches of seagrass that were similar in dimension to our ASUs. Each sample was fixed in a 10% formalin solution and stained with 0.2% Rose Bengal. Encrusting invertebrates were then manually removed while the remainder of the sample was sieved on 250 μm mesh and preserved in 70% ethanol.

At this stage the clean invertebrate samples from snapper gut contents, artificial and natural seagrass blades and sediment underwent a similar process to that of the zooplankton samples. Invertebrates were sorted, counted and identified down to the lowest practical taxonomic level.

### Data processing and statistical analysis

Invertebrate data from the five collection methods (zooplankton, gut contents, artificial seagrass blade, natural seagrass blade and sediment samples) were standardised to assist with comparison. For each collection method we first averaged the count of each taxonomic grouping at a site, and then across all sites (except natural seagrass samples, where we only sampled one site). All counts were then standardised by the most abundant taxonomic group.

For data obtained from video observations, each response variable was represented as the proportion of two minute video segments where a certain observation occurred. For abundance, post-settlement snapper were either present or absent, so the response variable was simply the proportion of times when post-settlement snapper were present. For location, the response variable was based on the most frequently occurring category (in this case the proportion of times when post-settlement snapper were observed near seagrass). For feeding location, nearly all observations were for feeding that occurred in the water column, so we constrained our analysis to data that reflected the proportion of time segments when post-settlement snapper were present that feeding occurred in the water column. The final response variable was also standardised for the influence of abundance by dividing by the post-settlement snapper presence variable above.

One way ANOVAs were used to test for significant differences between response variables. We treated sites as a fixed factor as we had intentionally selected sites based on their water velocity. Where needed, data were transformed (arcsine or square root) to meet assumptions of normality and homoscedasticity. Where statistically significant differences were detected, individual differences were identified using Student-Newman-Keuls *post hoc* tests. For one analysis (comparison of post-settlement snapper abundance obtained from ‘brush and dustpan’ netting) transformations were not able to improve heterogeneity of variance, so a one way non-parametric Kruskal-Wallis test was performed instead. Where linear regressions were performed we followed a similar process for assessing assumptions as described for ANOVAs above. Data used for these analyses and plots are provided (see S1 Data).

## Results

Due to the exploratory nature of this study we collected a diverse array of samples as well as over 40 hours of video footage containing post-settlement snapper. All of these samples were obtained within a range of water flow velocities that post-settlement snapper may experience while occupying estuarine habitats. Below we present results from these different methods in combined sections addressing three aspects of estuarine nursery habitat usage.

### Abundance and condition of post-settlement snapper near ASUs

**Fig 2:**
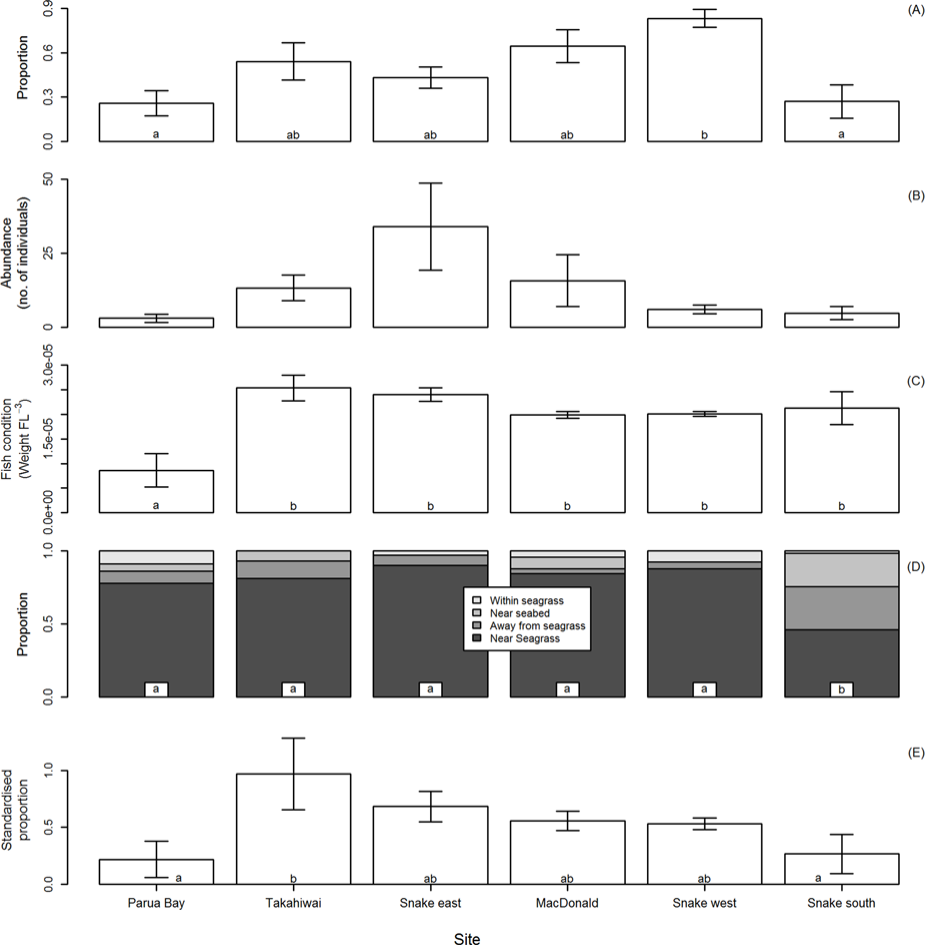
Abundance, condition, pelagic feeding frequency and location of post-settlement snapper by site as observed from video deployments and ‘brush and dustpan’ netting in Whangarei Harbour. (A) Abundance: proportion of two minute video segments where post-settlement snapper were observed. (B) Abundance: number of snapper captured by ‘brush and dustpan’. Kruskal Wallis test did not find any significant differences. (C) Fish condition: Condition of post-settlement snapper captured by ‘brush and dustpan’ netting. (D) Location: average proportion of observations of post-settlement snapper within each position category (see methods for category definitions). Statistical test conducted for observations that were categorised as near seagrass. (E) Feeding events: proportion of two minute video segments where post-settlement snapper were present that suspected pelagic feeding events were observed (standardised for snapper abundance). For all plots sites are listed in increasing order of maximum water velocity (from Whangarei Harbour hydrodynamic model), values at each site are averages from four replicate ASUs ±1 standard error. Different lowercase letters denote significant differences between sites at the α = 0.05 level (Student-Newman-Keuls tests).

When sites were ordered by increasing maximum current velocity, video observations of post-settlement snapper abundance initially increased, levelled off and eventually decreased (Fig. 2A). Abundance was significantly lower at sites with the lowest and highest maximum current velocities (Parua Bay and Snake Bank South) compared with one of the sites with intermediate maximum current velocity (Snake Bank West) (1-way ANOVA: *df* = 5, *F* = 5.22, *p* < 0.004). The abundance of post-settlement snapper captured by brush and dustpan netting demonstrated a hump-shaped pattern, initially increasing and then decreasing, across sites when ordered by increasing maximum current velocity (Fig. 2B). This dataset, however, contained heterogeneous variances between sites, which could not be corrected by transformation. The resulting non-parametric test did not detect any significant differences in abundance between sites (1-way Kruskal-Wallis test: *df* = 5, *Kruskal-Wallis statistic* = 8.94, *p* = 0.111).

The condition (weight × FL^-3^) of post-settlement snapper captured via brush and dustpan netting was similar between all sites except for the site with the lowest maximum current velocity (Parua Bay) (Fig. 2C). This site had a significantly lower average condition value compared to all other sites (1-way ANOVA: *df* = 5, *F* = 6.535, *p* = 0.001).

### Behaviour and predators of post-settlement snapper near ASUs

Only three species observed on the more than 40 hours of video footage containing post-settlement snapper were considered as potential predators (i.e. medium to large sized carnivores with a piscivorous component to their diet). These included one observation of an adult trevally (*Pseudocaranx georgianus*), 18 observations of broad squid (*Sepioteuthis australis*), and 128 observations of large (≥1+) snapper. None of these observations, however, included predation or attempted predation events.

Snapper were most frequently observed near the seagrass (i.e. within 30 cm; Fig. 2D), and were infrequently observed moving within the seagrass blades themselves. This trend was consistent for all but the site with the strongest maximum current strength (Snake Bank South). We tested this relationship by treating the proportion of observations near seagrass as a univariate response variable. Snake Bank South had a significantly lower proportion of observations where snapper were classified as near seagrass (1-way ANOVA: *df* = 5, *F* = 4.08, *p* < 0.012) with observations classified as away from seagrass or near seabed accounting for this difference.

### Diet, feeding and potential prey distribution of post-settlement snapper near ASUs

**Fig 3:**
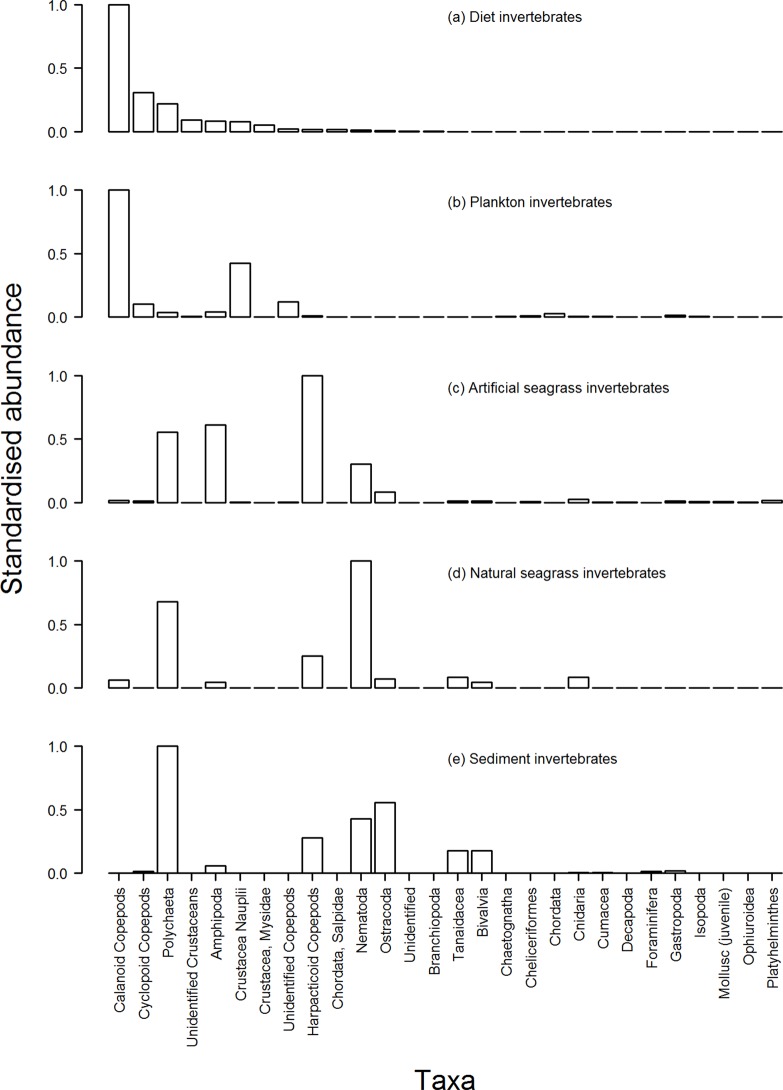
Abundance of invertebrates collected from all sites via four different sampling methods. Abundances are standardised by the most abundant taxa within a sampling method, but all plots are presented in order of abundance for diet invertebrates. Taxonomic nomenclature presented is not always at same level.

**Fig 4:**
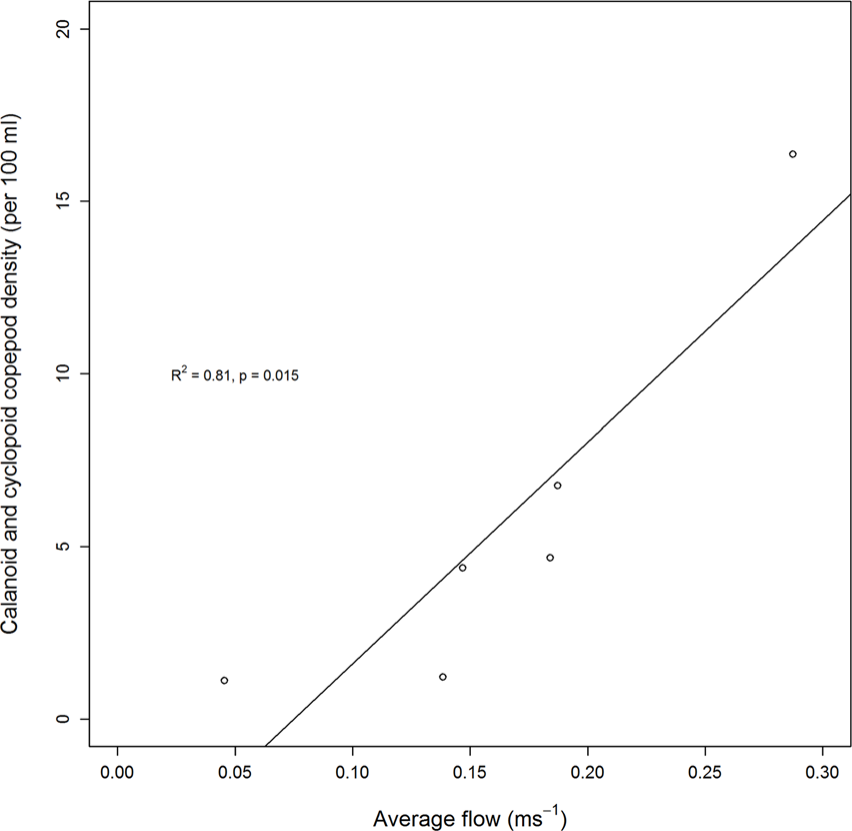
Relationship between water velocity and the density of post-settlement snapper prey in the water column. Water velocities are averages and derived from the Whangarei Harbour hydrodynamic model. Post-settlement snapper prey is represented by plankton sample abundances of the two most abundant taxa found in gut contents (calanoid and cyclopoid copepods).

The five sampling techniques utilised to investigate diet and invertebrate abundance within specific micro-habitats around ASUs identified 35 different invertebrate taxa from the thousands of invertebrates we collected and counted. Snapper gut samples were dominated by calanoid copepods, cyclopoid copepods, pieces of polychaete worms and unidentified crustaceans. Sediment samples were dominated by polychaete worms, ostracods, nematode worms and harpacticoid copepods. ASU blade samples were dominated by harpacticoid copepods, amphipods and polychaete and nematode worms. Natural seagrass blade samples were dominated by nematode worms, polychaete worms and harpacticoid copepods. Plankton samples were dominated by calanoid copepods, crustacean nauplii, unidentified copepods and cyclopoid copepods (Fig. 3). The influence of average current strength across sampling sites on the density of calanoid and cyclopoid copepods (identified as important diet items) within plankton samples was then assessed with linear regression (Fig. 4). Calanoid and cyclopoid copepods demonstrated an increasing trend in density as average current strength also increased (*p* = 0.015, *r* ^*2*^ = 0.81).

Video observations of suspected feeding events around ASUs were much more common in the water column than elsewhere (836 two minute video segments contained suspected feeding events in the water column vs 48 on the benthos or amongst seagrass blades). As a result we constrained the remainder of our feeding analysis to observations of suspected feeding in the water column. When only considering time segments when post-settlement snapper were present, the frequency of feeding events initially increased and then gradually decreased in frequency (across sites ordered by increasing maximum current velocity) (Fig. 2E). Feeding events were significantly less frequent at sites with the lowest and highest maximum current velocities (Parua Bay and Snake Bank South) compared with Takahiwai (1-way ANOVA: *df* = 5, *F* = 4.34, *p* < 0.009).

## Discussion

Unequivocally establishing the nursery value of a habitat is difficult [1], so it follows that understanding the mechanisms underlying that nursery function will also be challenging. This study employed an exploratory approach to address potential mechanisms that may explain the association of post-settlement snapper with structurally complex habitat. For example, more than 90 hours of video were collected from around ASUs (40 hours of which contained post-settlement snapper). One of the more conspicuous aspects of this footage was the almost complete absence of predators that may regulate an association between post-settlement snapper and structurally complex habitats. This could be because predation events only occur in deeper water, at night or in the absence of structure (and therefore our cameras). It seems likely that if predation were important, some interaction with potential predators would be observed around the edges of ASUs. While many previous studies have used techniques such as tethering to demonstrate reduced predation within seagrass [3], others have also found little difference in the survival of juvenile fishes between seagrass and bare habitats [19,20]. In some areas juvenile fishes even avoid seagrass due to the presence of ambush predators within the seagrass itself [21]. Overall, however, the way in which seagrass influences the survival of juvenile fishes is species specific, dependent on multiple factors including predator distribution and habitat patchiness [22], changing habitat requirements with ontogeny [23], and the trade-off between food abundance and predation risk [24].

Our sampling also addressed the diet of post-settlement snapper associated with structurally complex habitats by comparing what snapper had eaten with the availability of potential food sources in different micro-habitats around ASUs and amongst natural seagrass blades. While previous studies have indicated that copepods and other potentially pelagic crustaceans are part of the diet of post-settlement snapper [7], in the present study calanoid and cyclopoid copepods dominated post-settlement snapper diet. Most diet studies are prone to overestimating the importance of items with hard body parts [25]. This seems unlikely to be a major factor in the present study as digested matter and items that were digested to a state where they were no longer identifiable were not a major component of snapper gut contents. While a comparison of diet composition between sites may have been informative, it was precluded by low sample sizes for some sites. Another potential concern is that ASUs may have much lower prey availability compared to natural seagrass. This was not the case in the present study, with the most important snapper diet items being of similar or higher relative density (no. per plant) on ASUs compared to natural seagrass (calanoid copepods 0.86 vs. 3; cyclopoid copepods 0.85 vs. 0; polychaete worms 43 vs. 33; amphipoda 106 vs. 2).

In terms of the niche occupied by major diet items, calanoid and cyclopoid copepods are generally pelagic [26]. Furthermore, other invertebrate sampling conducted in the present study most frequently captured calanoid and cyclopoid copepods within the water column (i.e. plankton samples). In addition, video footage of suspected feeding events demonstrated that snapper feeding occurred almost exclusively within the water column. Together these results suggest that snapper are planktivorous during their post-settlement stage, before shifting to a diet dominated by benthic food items as larger juveniles and adults [6,7]. Similar ontogenetic shifts (pelagic to benthic prey) also occur for the juvenile stages of other coastal fish species [27–29], and may reflect an increase in available prey items with increasing jaw size.

When the planktivorous feeding described above is considered in combination with the lack of obvious predation threat, it is not immediately apparent how the nursery-role benefits of increased survival and elevated growth [1] would be conveyed to snapper through an association with seagrass. In other non-estuarine habitats, however, the interplay of water currents and zooplankton are better established [30–33]. With this perspective, some insight is provided when the velocity of tidally driven water flow is considered across our sites. Sites with faster water flow had more calanoid and cyclopoid copepods, while snapper abundance and observations of pelagic feeding events were lower at sites with the highest and lowest water flows. To establish these relationships we ordered sites by average flow for planktonic food abundance (because our samples were taken across a range of different tidal states potentially representing the average flow at each site) and maximum flow for the response of the fish themselves (we assumed that the suitability of a site would likely be determined by the ability of fish to maintain position at times of maximum flow). We had anticipated that the flux of copepods may be greater at high flow sites, as zooplankton flux is the product of prey density and current speed [18]. It is not clear, however, why the density of copepods was also higher for these sites, but lateral advection of plankton by water currents is known to be both highly variable and influential [34], and may result in the differential delivery of copepods to different parts of the Harbour. Regardless, the high copepod density that we observed in combination with high average water velocity infer increased availability of planktonic food items. In terms of snapper abundance, the significance of the hump-shaped relationship suggested by netting was precluded by high variation, potentially driven by the one off snapshot of abundance that netting provides. Video observations, where data were obtained across a number of different times and tidal states, confirmed that one of the sites with intermediate flow had higher abundance than the sites with the lowest and fastest flow. It could also be expected that the size of post-settlement snapper may be influenced by water velocity, with larger fish potentially being more capable of maintaining faster swimming speeds [35] required at high water velocity sites. This was not true for the present study; the site with the fastest water velocity (Snake Bank South) also had the smallest snapper size distribution (17.5 mm median FL).

Together these results suggest that during their post-settlement stage, the influence of water flow on the availability of zooplankton food is likely to be important to snapper. A potential explanation is that benefits are greatest at intermediate flow rates; a trade-off driven by low flux of zooplankton food at low flow and the increased cost of swimming [36] and/or decreased feeding efficiency that occurs through the narrowing of a fish’s visual reactive volume [18] at high flow. Alternatively, low abundance and condition at low flow sites could be driven through a response to turbidity [8] (which has the potential to be elevated when flow is low) or a decreased potential for snapper larvae to be delivered to those sites by their slower water currents. These alternatives, by themselves, do not explain why both the abundance and the frequency of pelagic feeding events were lowest at both the low and high flow sites.

While the interplay between water flow and zooplankton is likely an important driver for post-settlement snapper, this does not explain why post-settlement snapper are so closely associated with structurally complex habitats [8]. We had hoped that video deployments would provide more insight here, but cameras had to be placed very close to ASUs to obtain sufficient resolution to observe post-settlement snapper, which resulted in a restricted field of view (relative to the whole ASU). What these observations did show, however, was that post-settlement snapper were generally near the seagrass, as opposed to within it or well away from it. While it is possible that the proportion of snapper within the seagrass was underestimated due to obscured vision, this seems unlikely. Juvenile snapper rarely remain stationary resting on the seabed (D. Parsons pers. obs.), hence within two minute observations we would likely see juvenile snapper leaving or entering the seagrass if they often resided between the seagrass blades themselves. It is not clear, however, why this relationship differed for the highest flow site, but this may reflect a change in feeding and/or refuging mode at higher flow rates. We do know that significant reductions in flow speed occur around the fringes of seagrass and other permeable canopies (including locations above as well as down and upstream relative to a patch) [37–41] and that reduced flow speeds around structures are used as energetic refuges by fishes [42]. Therefore, it is possible that structurally complex habitats may provide post-settlement snapper with an energetic refuge at sites with higher flow and therefore higher food availability, as has been demonstrated for other fish species [9,36,43]. Finer—scale behavioural observations and current readings relative to structure are required to confirm the importance of seagrass as an energetic refuge for post-settlement snapper.

While nursery habitat benefits, such as protection from predation, are well established [3,44], many nursery occupying, fish species also have a planktivorous component to their diets [45–49]. Furthermore, the importance of the interaction between planktivorous feeding and swimming costs in high flow environments is well established for non-estuarine fish species [9,36,43]. This information, and the findings of the present study, are all compatible with the possibility that part of the value of nursery habitats may arise through how they contribute to planktivory and energetic refuging in high flow environments. For snapper, the identification and management of the most productive nursery habitats has been identified as a key priority [5]. Our results around water velocity provide context to this statement; all structurally complex habitats are unlikely to have equal value to post-settlement snapper. As such, predictive models combining current velocity and other physical variables that are important to snapper [8] may have great utility in identifying and directing management resources to the most valuable nurseries.

## Supporting Information

S1 Data Data used in analyses and plots for ‘brush and dustpan’ netting of snapper abundance, invertebrate sampling of diet/ potential diet items, and video observations of snapper behaviour and abundance. (TXT)
